# Author Correction: Full-gap superconductivity in spin-polarised surface states of topological semimetal *β*-PdBi_2_

**DOI:** 10.1038/s41467-017-02137-4

**Published:** 2017-12-14

**Authors:** K. Iwaya, Y. Kohsaka, K. Okawa, T. Machida, M. S. Bahramy, T. Hanaguri, T. Sasagawa

**Affiliations:** 1 RIKEN Center for Emergent Matter Science, Wako, Saitama 351-0198 Japan; 2 Laboratory for Materials and Structures, Tokyo Institute of Technology, Yokohama, Kanagawa 226-8503 Japan; 3 Department of Applied Physics, The University of Tokyo, Hongo, Bunkyo-ku, Tokyo 113-8656 Japan

*Nature Communications* **8**:976 10.1038/s41467-017-01209-9; Article published online: 17 October 2017

This Article contains an error in Fig. 3 in that the calculated patterns of quasiparticle interference in the figure are incorrect due to the wrong Wannier transformation in the calculation process. The correct version of Fig. 3 appears below. This correction does not affect the discussion or the conclusion of the Article.

**Fig. 3:**
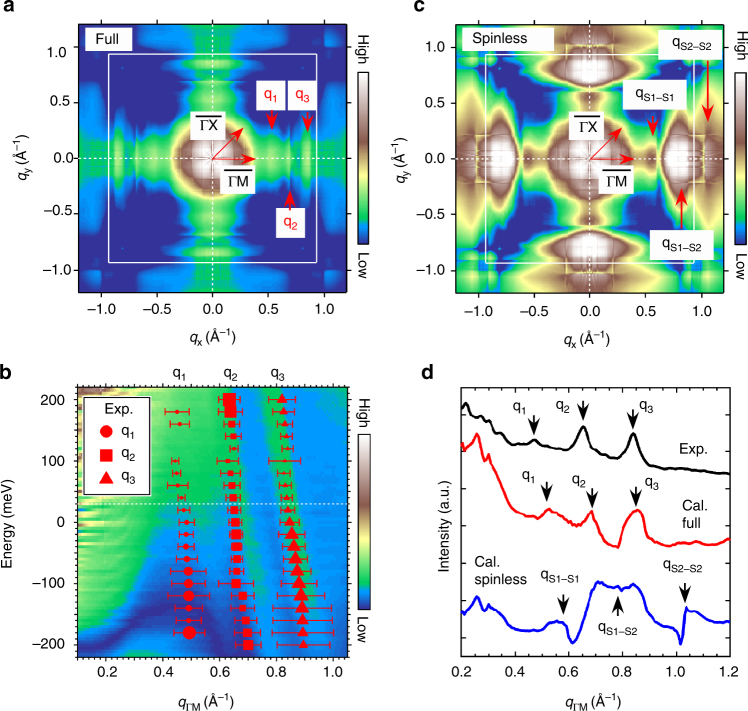
Fig.3

