# Supplementary material for: Full-gap superconductivity in spin-polarised surface states of topological semimetal β-PdBi2
Source: Nat Commun. 2017 Oct 17;8:976. doi: 10.1038/s41467-017-01209-9 (PMC5730620; doi:10.1038/s41467-017-01209-9)
Supplement: Supplementary file 1 — Supplementary Information [file 41467_2017_1209_MOESM1_ESM.pdf]

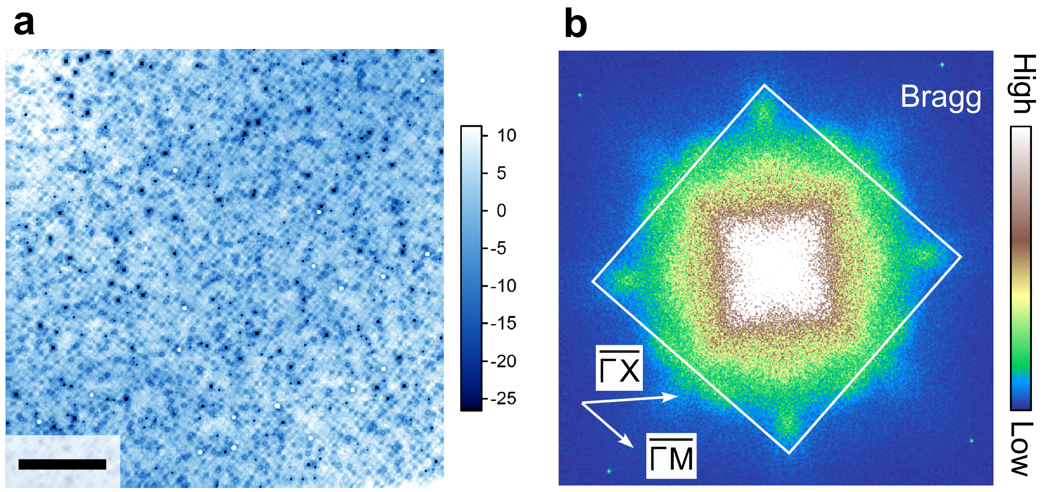

Supplementary Figure 1: **Atomically-flat β-PdBi$_2$ surface.** (**a**) A constant-current STM image of β-PdBi$_2$. 100×100 nm^2, $V = +200$ mV, $I = 200$ pA. The scale bar corresponds to 20 nm and the colour scale is in pm. (**b**) Fourier-transformed image of **a**. Bragg peaks corresponding to $a_0 = 0.337$ nm are clearly seen. The image is symmetrized with respect to the four-fold symmetry of the crystal structure. The first Brillouin zone is indicated by a white square.

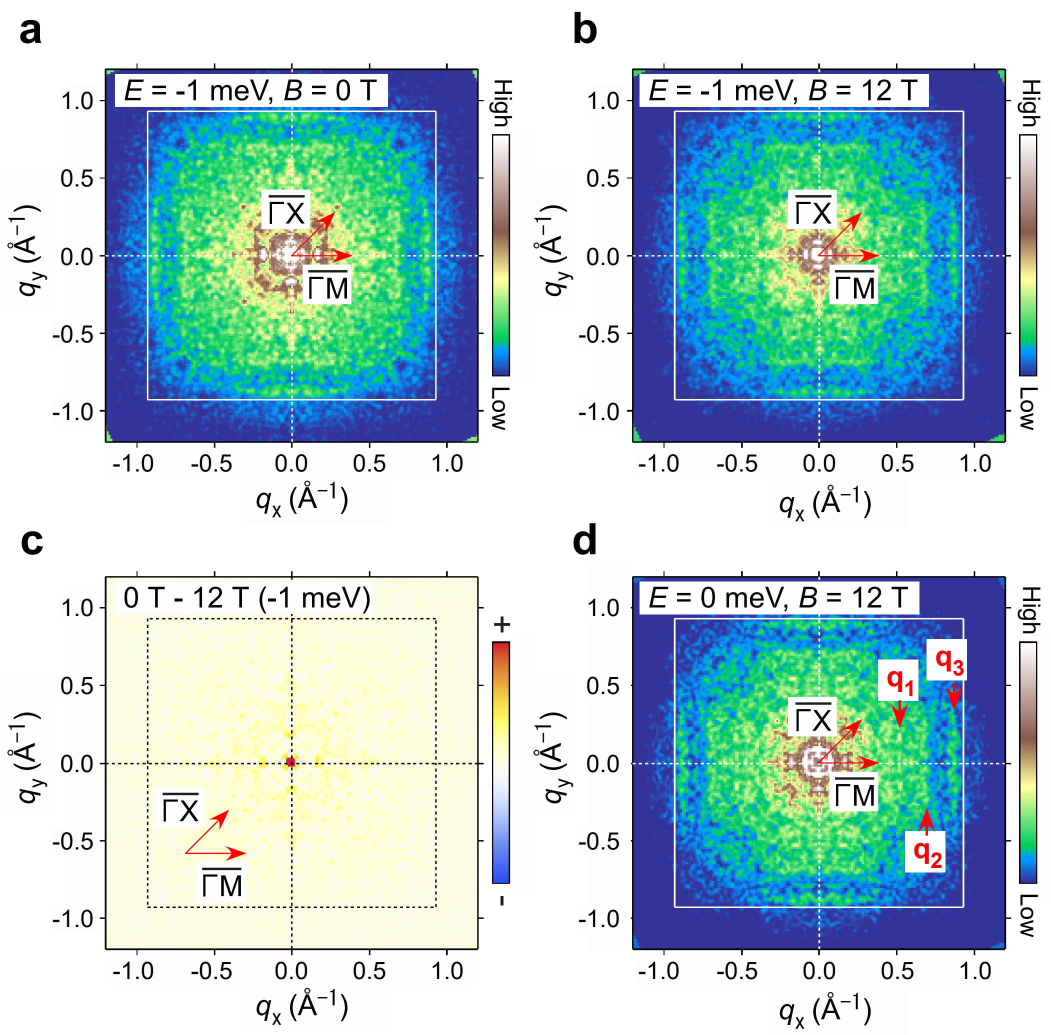

Supplementary Figure 2: **QPI patterns in the SC state and the normal state near E_{F}.** (**a,b**) Fourier-transformed image of dI/dV map at $V = -1$ mV in zero field and $B = 12$ T, respectively. $T = 1.5$ K. Both images were taken in the same field of view. Setup conditions for imaging were $V = +10$ mV, $I = 100$ pA. The bias voltage was modulated at 617.3 Hz with an amplitude V_{rms} of 350 μV. The image is symmetrized with respect to the four-fold symmetry of the crystal structure. The first Brillouin zone is indicated by a white square. (**c**) Difference between **a** and **b**. No clear QPI signals are found except at $\boldsymbol{q} = 0$. (**d**) Fourier-transformed image of dI/dV map at $V = 0$ mV in $B = 12$ T. $T = 1.5$ K. All of the setup conditions are the same as in **a** and **b**. The four-fold QPI pattern indicates that the spin-polarized states discussed in the main text indeed exist at E_{F} in the normal state.

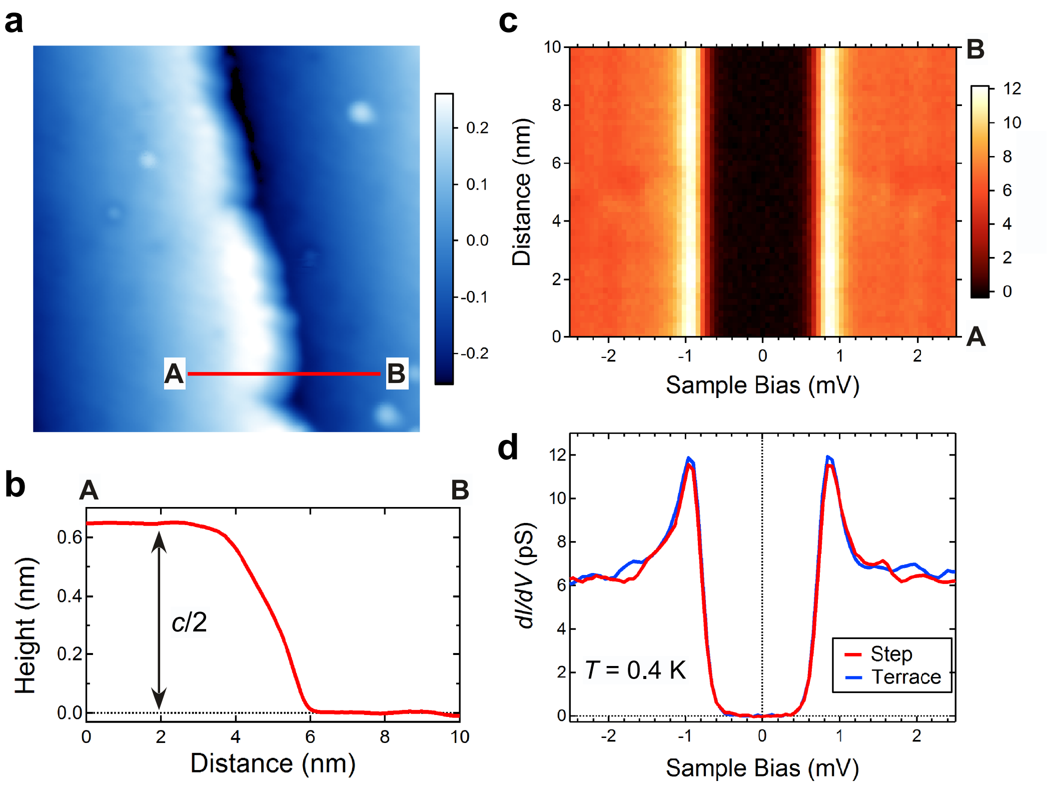

Supplementary Figure 3: **Absence of a zero-bias conductance peak at the step edge.** (**a**) A constant-current STM image across the step structure. 20×20 nm^2, $V = +90$ mV, $I = 10$ pA. $T = 0.4$ K. The colour scale is in nm. (**b**) A line profile across the step structure in **a**. The step height corresponds to $c/2 = 0.65$ nm. (**c**) dI/dV spectra across the step structure at $T = 0.4$ K. Setup conditions: $V = +15$ mV, $I = 100$ pA. Lock-in parameters: $V_{\mathrm{rms}} = 42$ μV, $V_{\mathrm{freq}} = 617.3$ Hz. The colour scale is in pico siemens (pS). (**d**) dI/dV spectra at the step edge and on the terrace in **c**.

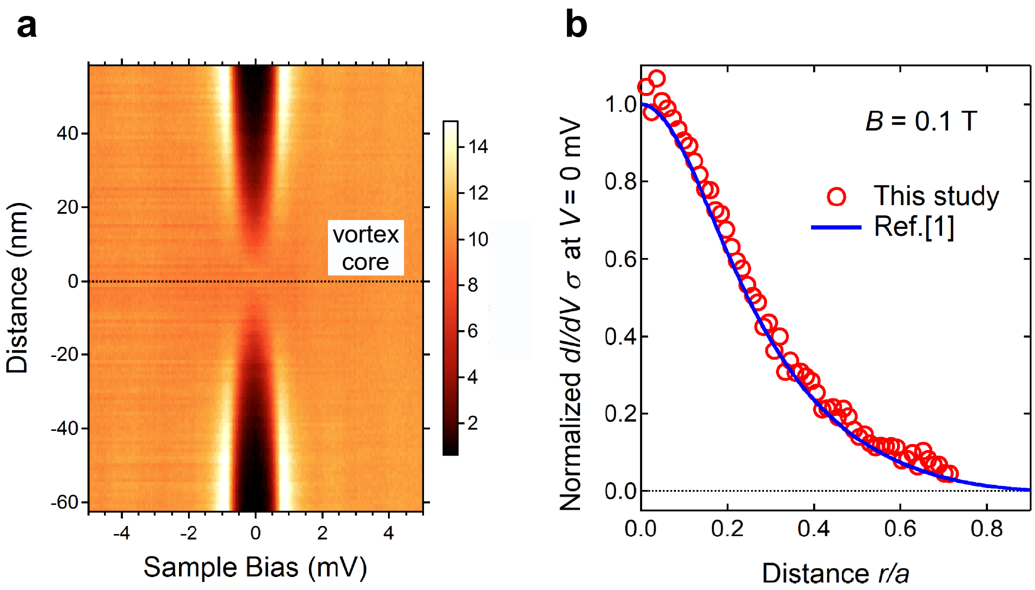

Supplementary Figure 4: **Line profile of a vortex core.** (**a**) dI/dV spectra across the vortex core. $T = 0.4$ K and $B = 0.1$ T. Setup conditions: $V = +10$ mV, $I = 100$ pA. $V_{\rm rms} = 35$ μV. The colour scale is in pico siemens (pS). (**b**) Normalized zero bias conductance line profile in **a**. The distance from the vortex core is normalized by $a = 81.2$ nm at $B = 0.1$ T, defined as $\pi a^2 = \phi_0/B$ where ϕ_0 is the flux quantum. For comparison, the line profile reported in ref. [1] ($B = 0.1$ T, $T = 0.15$ K) is also plotted.

Supplementary Note 1 - The set-point effect in Fourier-transformed dI/dV images

The tunnelling current $I(\mathbf{r}, z, V)$ at low temperatures using a typical metallic tip can be generally described as

$$I(\mathbf{r}, z, V) \propto \exp(-2\kappa z) \int_0^{eV} \rho_s(\mathbf{r}, E) dE \qquad (1)$$

where $\mathbf{r}$, z, V, κ and ρ_s is the lateral location on the sample surface, the tip-sample distance, the bias voltage applied to the sample, the decay length inverse and the local density of state of the sample, respectively. By differentiating this tunnelling current with respect to V, we obtain the differential conductance:

$$\frac{\partial I}{\partial V}(\mathbf{r}, z, V) \propto \exp(-2\kappa z)\rho_s(\mathbf{r}, eV) \qquad (2)$$

In the constant current mode, the tunnelling current is regulated to be constant by the feedback loop, which is opened during the spectroscopic measurement, so that Supplementary Eq.(2) can be rewritten as,

$$\frac{\partial I}{\partial V}(\mathbf{r}, V, V_{\text{set}}, I_{\text{set}}) \propto \frac{I_{\text{set}}\rho_s(\mathbf{r}, eV)}{\int_0^{eV_{\text{set}}} \rho_s(\mathbf{r}, E) dE} \qquad (3)$$

where V_{set}, I_{set} is the set-point bias voltage and the set-point current, respectively. The denominator of Supplementary Eq.(3) represents the set-point effect [2].

To examine the set-point effect on Fourier-transformed QPI images, let us consider to integrate the QPI signal at a wavevector $\mathbf{q}$ with respect to the bias-voltage from 0 to V_{set} as follows:

$$\int_0^{V_{\text{set}}} \Big[\int \frac{\partial I}{\partial V} \exp(-i\mathbf{q}\cdot\mathbf{r})d\mathbf{r}\Big] dV = \int \exp(-i\mathbf{q}\cdot\mathbf{r})d\mathbf{r} \int_0^{V_{\text{set}}} \frac{\partial I}{\partial V} dV = I_{\text{set}}\delta(\mathbf{q}) \qquad (4)$$

This means that the integration from 0 to V_{set} will be always zero at $\mathbf{q} \neq 0$. To satisfy this requirement, each Fourier component except at $\mathbf{q} = 0$ will change its sign as a function of V and should become zero at least once between 0 and V_{set}. The suppression of QPI signals at around +100 mV in Fig. 2d in the main text is due to this zero-crossing.

Supplementary References

[1] Fente, A *et al.* Field dependence of the vortex core size probed by scanning tunneling microscopy. *Phys. Rev. B* **94**, 014517 (2016).

[2] Kohsaka, Y *et al.* An intrinsic bond-centered electronic glass with unidirectional domains in underdoped cuprates. *Science* **315**, 1380-1385 (2007).
